# Development of a rapid, efficient, and reusable magnetic bead-based immunocapture system for recombinant human procollagen type II isolation from yeast fermentation broth

**DOI:** 10.1007/s00216-023-04752-1

**Published:** 2023-05-29

**Authors:** Martina Lioi, Sara Tengattini, Francesca Bagatin, Stefano Galliani, Simona Daly, Gabriella Massolini, Caterina Temporini

**Affiliations:** 1 Department of Drug Sciences, University of Pavia, Viale Taramelli 12, 27100 Pavia, Italy; 2 Gnosis By Lesaffre, Via Lavoratori Autobianchi 1, 20832 Desio, Italy

**Keywords:** Magnetic beads, Antibody, Immunocapture, Recombinant human type II procollagen, Yeast fermentation

## Abstract

Recombinant collagen production, especially using yeasts as expression systems, could represent a promising alternative over traditional extractive methods from animal sources, offering controllable, scalable, and high-quality products. Monitoring the efficiency and efficacy of procollagen/collagen expression, especially in the initial fermentation phases, can be difficult and time consuming, as biological matrices necessitate purification and commonly used analytical methods are only partially informative. We propose a straightforward, efficient, and reusable immunocapture system able to specifically isolate human procollagen type II from fermentation broths and to release it in few experimental steps. A recovered sample allows for a detailed characterization providing information on structural identity and integrity, which can strongly support the monitoring of fermentation processes. The immunocapture system relies on the use of protein A–coated magnetic beads which have been functionalized and cross-linked with a human anti-procollagen II antibody (average immobilization yield of 97.7%) to create a stable and reusable support for the specific procollagen fishing. We set up the binding and release conditions ensuring specific and reproducible binding with a synthetic procollagen antigen. The absence of non-specific interaction with the support and binding specificity was demonstrated, and the latter was also confirmed by a peptide mapping epitope study in reversed-phase liquid chromatography high-resolution mass spectrometry (RP-LC-HRMS). The bio-activated support proved to be reusable and stable over 21 days from the initial use. Finally, the system was successfully tested on a raw yeast fermentation sample to provide a proof of concept of the applicability within recombinant collagen production.

**Graphical Abstract:**

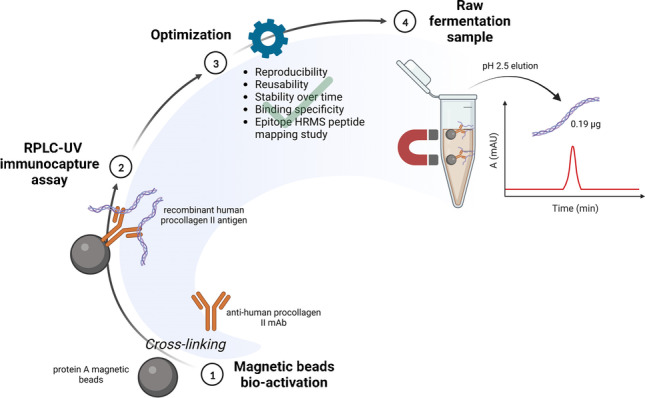

## Introduction

Collagen is a structural protein that plays a key role in the biopharmaceutical industry. As a major component of skin, tendons, and bones, collagen has a wide range of potential therapeutic applications, from wound healing to tissue engineering [1–3]. Collagen is traditionally derived from animal sources with limitations and drawbacks, including the ethical and environmental concerns associated with the use of animal products, in addition to the risk of transmitting highly infective diseases, and potential antigenicity. Moreover, the variability in the quality and quantity of collagen obtained from different animal sources is an additional concern [4–6].

The production of recombinant collagen using biotechnology techniques could result in controllable, scalable, and sustainable processes, leading to homogeneous products, free from contaminants. Several prokaryotic (bacteria) and eukaryotic systems (yeasts, mammalian and insect cells, plants) have been explored to produce recombinant collagen, with yeast being at present the most effective expression system [7, 8]. Recombinant technology may overcome most limitations of extractive methods, making it an attractive alternative for the biopharmaceutical industry [9–11]. However, this way of production is still in its early stages due to collagen intricate biosynthesis [7].

Collagen is initially produced as procollagen, with two additional fragments, the N-terminal and C-terminal propeptides, over the central portion represented by the alpha chain. Propeptides participate in the polymerization of three alpha chains to form a collagen triple helical domain, and then they must be removed by metalloproteinases before the formation of more complex fibers [12, 13]. In addition, to attain its native form during biosynthesis, collagen necessitates the incorporation of post-translational modifications such as the hydroxylation of proline, which is catalyzed by the enzyme prolyl 4-hydroxylase (P4H), leading to the formation of 4-hydroxyproline. This modified amino acid is a prerogative of collagen and is fundamental for the proper folding and stability of the triple helix [14]. While mammalian cells possess the complete post-translational machinery required for accurate and functional collagen production, insect and yeast cells have limited or nonexistent P4H activity, respectively. Therefore, it is mandatory to insert in these expression systems not only the gene encoding the alpha chain, but also the enzymes responsible for the protein biological activity [7].

Methods for identification and quantification of collagen involve a wide variety of both traditional and next-generation approaches, each with its own advantages and disadvantages in terms of sensitivity, specificity, and operating time. The hydroxyproline measurement employing collagen hydrolysis and a derivatization reaction followed by HPLC–MS detection of the released amino acids is widely regarded as the gold standard, as it offers a general estimation of collagen content [15, 16]. However, this method lacks selectivity and requires further analysis for detailed collagen characterization. SDS-PAGE is commonly used to detect collagen by separating alpha chains based on molecular weight but is not particularly sensitive or selective for closely related collagen forms, necessitating additional identification techniques. ELISA-based assays offer greater specificity for different types of collagens, but they do not provide information about structural integrity. An exhaustive overview of available analytical and biochemical methods is reported by Bielajew et al.[17].

A critical aspect in the expression of recombinant procollagen alpha chains deals with monitoring the integrity and stability of the protein during fermentation, which can be affected by the presence of proteases in the fermentation media, by downstream processing, or by issues with the expression strain engineering. Regardless of the analytical method adopted, the preparation of samples for analysis can be difficult and time consuming due to the complexity of the biological matrix in which collagen is found. Thus, there is the need for a rapid and effective method to isolate procollagen from the cultured medium and to verify its integrity as a direct proof of the expression efficiency.

Immunoaffinity systems may represent a potential solution. By exploiting a highly specific interaction such as the one between antibodies and their antigens, these methods can provide a quick and efficient means of monitoring protein production when coupled to other analytical techniques (e.g., mass spectrometry, ELISA) to accurately characterize and quantify protein levels. One of the main advantages of these systems is their high specificity and selectivity, allowing for efficient purification of the target protein from complex mixtures, providing a high-quality product [18]. Among the many solid supports that are used for the placement of the immunocapture agent, both organic and inorganic, the use of magnetic beads must be highlighted. Bead-based supports offer several benefits for the purification of recombinant proteins, including high binding capacity, fast and easy separation from a solution using a magnetic field, and the ability to shorten different purification steps. This results in reduced processing time, as well as improved yields and purity of the final product [19–21].

In this article, we report the development of a bead-based immunoaffinity system that is fast, sensitive, and reusable. The system is designed to selectively isolate human procollagen type II from complex fermentation broths, allowing for its subsequent structural analysis. This could represent a valuable tool in monitoring the expression efficiency during the early stages of fermentation processes.

Protein A–coated magnetic beads were selected as solid support for the immobilization of human anti-procollagen II antibody, given their high grafting efficiency [22]. In order to create a stable and reusable support, a covalent bond was established between protein A and the antibody by promoting a cross-linking reaction [23]. Experimental conditions for the immunocapture assay were assessed using a synthetic antigen containing the N- and C-termini of human type II procollagen (Gln26-Ala181 and Ala1241-Leu1487), and then applied to a complex matrix derived from a yeast-based fermentation process. This system has been tested for selectivity and specificity using blank beads and a protein mixture (antigen, bovine serum albumin), and in terms of binding capacity, reusability, and stability. Finally, the specificity of the binding has been empirically demonstrated by a peptide epitope mapping study.

## Materials and methods

### Chemicals and reagents

Protein A magnetic beads (1 mL) were purchased from New England Biolabs (Ipswich, MA, USA). HPLC-grade glycine, dimethyl pimelimidate dihydrochloride, triethanolamine hydrochloride, ethanolamine hydrochloride, TWEEN**®** 20 for molecular biology, and bovine serum albumin lyophilized powder were from Merck KGa (St. Louis, MO, USA). Phosphate-buffered saline (PBS, 10 ×) was from Thermo Fisher (Kandel, Germany). Recombinant human procollagen II, carrier free (20 μg) and human procollagen II antibody (100 μg) were provided by Bio-Techne (Minneapolis, MN, USA). Water was obtained from a Direct-QTM Millipore system (Millipore, Billerica, MA, USA), while acetonitrile (ACN) for HPLC–UV and trifluoroacetic acid (TFA) 99% were purchased from PanReac AppliChem ITW Reagents (Cinisello Balsamo, Italy). Tris base, sodium dihydrogen phosphate, sodium azide, dithiothreitol (DTT), and formic acid (FA) for mass spectrometry were from Merck KGaA (Darmstadt, Germany). Sequencing-grade modified trypsin was purchased from Promega Corporation (Madison, WI, USA).

### Apparatus

LC-UV separations were performed on Agilent HPLC series 1100 and 1200 systems (Santa Clara, CA, USA), equipped with a mobile-phase online degasser, a quaternary pump, an autosampler, a column thermostated compartment, and a diode array detector, and controlled by an Agilent ChemStation software (Rev. B.04.01).

For the epitope peptide mapping study, the chromatographic separations were performed on an ExionLC system (SCIEX, Framingham, MA, USA) equipped with a mobile-phase online degasser, a quaternary pump, an autosampler, and a column thermostated compartment. MS detection was carried out by an X500 QTOF mass spectrometer (SCIEX) with an ESI source. The LC–MS system was controlled by a SCIEX OS software (1.7 version).

### Immobilization and cross-linking of anti-human procollagen II IgG onto protein A magnetic beads

Anti-human procollagen II monoclonal antibody was grafted and cross-linked on the solid support by adapting commercial protocols for protein A magnetic beads. Twenty micrograms of human IgG was used to bio-functionalize 100 μL of beads. The antibody was reconstituted at 0.67 μg/μL in PBS 1 × .

Protein A magnetic beads were vortexed and resuspended. One hundred microliters of bead suspension was aliquoted to 1.5-mL microcentrifuge tubes. For two times, 500 μL of 0.1 M sodium phosphate buffer, pH 8.0 (binding buffer) was added to the beads and they were vortexed to be resuspended. By applying a magnet for 30 s, the beads were pulled to the side of the tube and the supernatant removed. Eighty microliters of binding buffer and 30 μL of 0.67 μg/μL IgG solution were added to the beads, mixed thoroughly, and incubated at 4 °C on a tube roller mixer (LLG-uni*ROLLER* 6 pro, Lab Logistics Group GmbH, Germany) for 30 min. The beads were separated with the magnetic device, and the supernatant was collected for the analysis. Beads were washed three times with 500 μL of binding buffer and supernatants from every washing step analyzed. A solution of 0.18 μg/μL of IgG in binding buffer was analyzed as the *t*_0_ to mimic the IgG concentration applied to the beads.

One milliliter of 0.2 M triethanolamine, pH 8.2 (cross-linking buffer) was added to the beads and the suspension was gently vortexed to resuspend. The supernatant was then removed by pulling the beads to the side of the tube with the magnet. This procedure was repeated twice. The beads were then resuspended thoroughly with 1 mL of cross-linking buffer containing 25 mM dimethyl pimelimidate (DMP) and incubated at room temperature for 45 min on the tube roller mixer. The magnet was applied, and the supernatant was discarded. One milliliter of 0.1 M of ethanolamine, pH 8.2 (blocking buffer) was added to the beads, and the suspension was gently vortexed to resuspend. The magnet was applied and the supernatant removed. One milliliter of blocking buffer was added and the suspension gently vortexed; the suspension was incubated for 30 min at room temperature under agitation. The supernatant was removed using the magnet. Three washing steps with 1 mL of PBS 1 × were performed and the supernatants discarded. To elute bound antibodies that were not cross-linked with DMP, 1 mL of 0.1 M glycine–HCl, pH 2.5 (elution buffer) was added to the beads and the suspension gently vortexed to resuspend. The suspension was incubated at 4 °C for 15 min under stirring. The supernatant was then collected for the analysis. This step was repeated to confirm the efficacy of cross-linking. The beads were then washed three times with 500 μL of PBS 1 × and the supernatants discarded. At this point, bio-functionalized magnetic particles ready to be used were resuspended and stored in 100 μL PBS 1 × , 0.1% Tween 20, and 0.02% NaN_3_ (storage buffer).

### Determination of the immobilization yield of IgG by high-performance size-exclusion chromatography (SEC-UV)

The monitoring of anti-procollagen II antibody immobilization and cross-linking was made by SEC-UV analysis of supernatants. The analyses were carried out using a TSKgel SuperSW3000 (4.6 mm ID × 300 mm L, 4 μm) from Tosoh Bioscience GmbH (Griesheim, Germany). Analytical conditions were set according to the producer’s recommendations: mobile phase 0.1 M Na_2_SO_4_, 0.05% NaN_3_ in 0.1 M sodium phosphate buffer, pH 6.7; flow rate 0.35 mL/min; and column temperature maintained at 25 °C. The injection volume was 5 μL and UV detection was carried out at 280 nm.

### Antigen capture and release assay with bio-functionalized protein A magnetic beads

Twenty-five microliters of bio-functionalized beads were pulled on the magnet, and the storage buffer was removed. After being washed twice with 500 μL PBS 1 × , the beads were incubated with 50 μL of 0.04 μg/μL recombinant procollagen II antigen (r-Ag) in PBS 1 × for 1 h at 4 °C on the tube roller mixer. Then, the magnetic beads were retained by applying the magnet and the supernatant collected for RP-LC-UV analysis. Three washing steps of 500 μL of PBS 1 × were required, and the supernatants were analyzed. Two release steps were finally performed by adding 50 μL of 0.2 M glycine–HCl, pH 2.5, and by incubating the solution for 15 min at 4 °C on the tube roller mixer. After incubation, the supernatants were collected for the analysis. Fifty microliters of the initial 0.04 μg/μL r-Ag solution was kept for 1 h at 4 °C under stirring and analyzed as the *t*_0_.

### RP-LC-UV analysis of supernatants from recombinant antigen binding and release cycles

An RP-LC-UV method was developed to analyze the r-Ag at the *t*_0_, supernatants from antigen binding and elution processes, and washing steps. An AdvanceBio RP-mAb C4 column (2.1 × 50 mm, 3.5 μm) from Agilent Technologies (Santa Clara, CA, USA) was selected using 0.1% v/v TFA in water (A) and 0.1% v/v TFA in ACN (B) as mobile phases and a flow rate of 0.3 mL/min. The elution program entailed 2 min of isocratic condition at 20% B followed by a linear gradient from 20 to 60% B in 8 min. The column was thermostated at 65 °C, and an injection volume of 10 μL was set. Samples were detected at 214 nm. A calibration curve was constructed using a solution of r-Ag with a concentration of 0.04 μg/μL in PBS 1 × . A range of dilutions of the r-Ag were made, from 0.02 to 2 μg, and analyzed to establish the calibration curve.

### Preparation of yeast fermentation broth samples containing r-Ag

Twenty milligrams of a lyophilized sample prepared from the fermentation broth of a wild-type yeast strain was solubilized in 4 mL of PBS 1 × , centrifuged for 5 min at 4 °C, 500 RCF, and filtered with 0.80 μm Mustang Q XT Acrodisc syringe filters (Pall Corporation, NY, USA). A 0.04-μg/μL solution was made by adding r-Ag in it (sample A). Sample B was obtained in the same way but without centrifugation and filtration, to maintain its raw conditions.

### Epitope peptide mapping study by RP-LC-HRMS

Digestion was carried out by preparing 100 μL of a 0.04-μg/μL solution of r-Ag with Tris–HCl, pH 7.8, and 1:10 v/v of DTT 0.5 M in water. This solution was firstly incubated at 60 °C for 30 min. 1:20 w/w trypsin was added, and the mixture incubated for 20 h at room temperature on the tube roller mixer. Fifty microliters was then incubated with 25 μL of bio-functionalized beads for 1 h at 4 °C under agitation following the already described protocol. Three washing steps were performed with 500 μL of PBS 1 × , and two elution steps under acidic conditions were repeated. Supernatants from all steps were recovered by applying the magnet and samples analyzed with RP-LC-HRMS. Fifty microliters of the digested solution was maintained under stirring at 4 °C for 1 h as well and analyzed as a reference.

RP-LC separations were carried out using a Kinetex® 5 μm EVO C18 100 Å (100 × 2.1 mm) column from Phenomenex (Torrance, CA, USA) and a mobile phase composed of H_2_O + 0.1% FA (A) and ACN + 0.1% FA (B). The elution program included 3 min of isocratic condition at 2% B followed by a linear gradient from 2 to 45% B in 57 min. The flow rate was set at 0.3 mL/min, and the column temperature was maintained at 60 °C. The injection volume was 20 μL.

The MS parameters applied in this study were as follows: positive mode, curtain gas pressure of 30 psi, ion source gas 1 pressure of 50 psi, ion source gas 2 pressure of 55 psi, temperature set at 350 °C, positive polarity, ion spray voltage of 5500 V, CAD gas of 7, 6 time bins were summed, TOF start mass set at 250 Da, TOF stop mass set at 2000 Da, accumulation time of 0.15 s, declustering potential of 80 V, and collision energy of 10 V. The experimental data were processed using the Explorer for SCIEX OS software (SCIEX) by comparing them with the r-Ag amino acid sequence in FASTA format. Peptides were selected based on an approximate LC peak width of 20 s, a minimum intensity of 3 counts, a chemical noise intensity multiplier of 1.5, a mass tolerance of 10 ppm, a maximum charge of 4, and maximum missed cleavages of 5.

## Results and discussion

In this work, an immunoaffinity system that uses magnetic particles functionalized with an anti-human procollagen type II IgG was developed. The system enables selective extraction of human procollagen type II from fermentation broths or cell lysates, allowing for the monitoring of procollagen characteristics (i.e., integrity, stability, degradation) during production. The rationale for this work is to support the production of recombinant human collagen by reducing the number of handling steps required for product analysis during fermentation processes. The specific isolation of procollagen type II carried out with this method could allow quickly determining whether its primary chain has been fully produced or whether it has been degraded during the expression/isolation phases.

Magnetic particles coated with protein A were selected as solid support for the immobilization of anti-procollagen II IgG through its Fc section. Additionally, the binding between the IgG and protein A was stabilized with cross-linking, thus leading to the creation of a reusable immunocapture system. Procollagen “fishing” and release phases were set up using a recombinant procollagen type II antigen. Hence, the proof of concept of the practical applicability of the bio-functionalized material was performed using a raw sample from a wild-type yeast strain, which was supplemented with the r-Ag, thus simulating a real fermentation sample.

As the IgG supplier does not provide specific information about the epitope region of the r-Ag–mAb complex, to validate the model used to set up the binding method, we performed an HRMS-based assay, for a direct proof of the propeptide region involved in the binding.

### Anti-human procollagen type II IgG immobilization and cross-linking

We initially developed an efficient immunoaffinity system that utilizes magnetic beads in miniaturized capture volumes (50 μL) able to not only detect the presence of procollagen in fermentation broths, but also isolate it for a comprehensive structural characterization. The first step involved the bio-functionalization of protein A–coated magnetic beads with the anti-human procollagen II IgG and cross-linking as described in “Determination of the immobilization yield of IgG by high-performance size-exclusion chromatography (SEC-UV).”

**Fig. 1:**
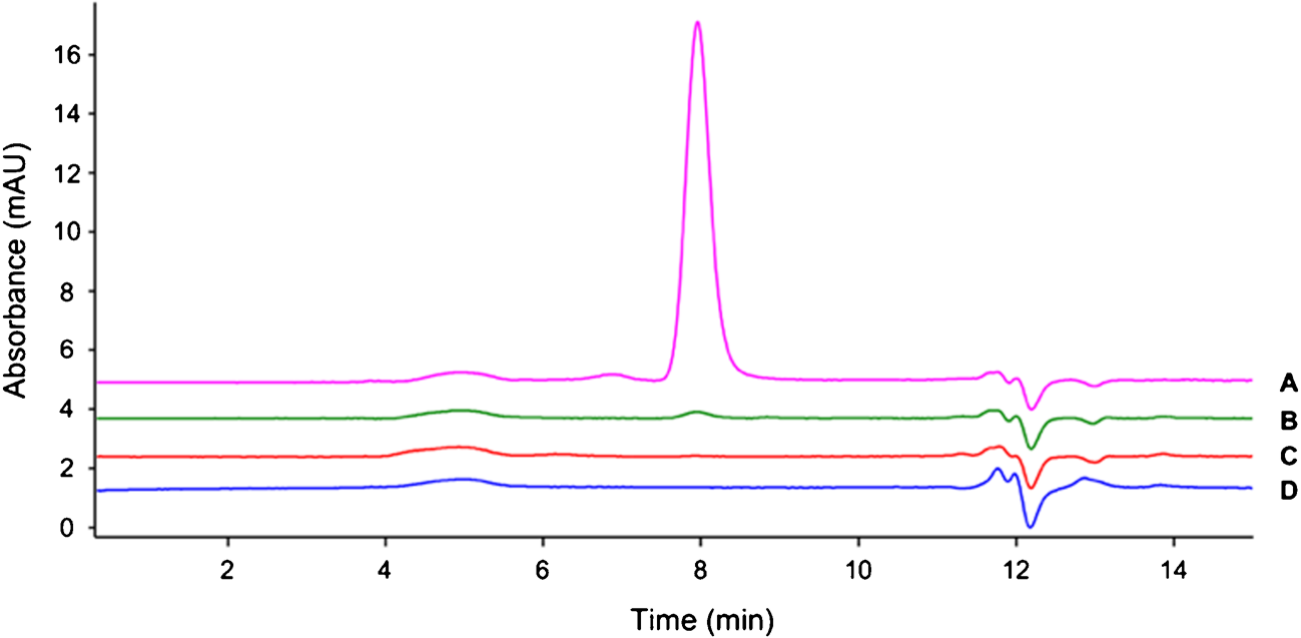
SEC-UV chromatograms of supernatants recovered during bio-activation of the beads with anti-human procollagen II IgG. **A**, pre-incubation IgG solution. **B**, supernatant analyzed after incubation for 30 min at 4 °C under stirring. **C**, supernatant analyzed after the first washing step (500 μL of 0.1 M Na phosphate buffer, pH 8.0). **D**, elution (step 1) of IgG not cross-linked promoted with 1 mL of 0.1 M glycine–HCl, pH 2.5, for 15 min at 4 °C

Immobilization yields (%) of the IgG bound to the beads were estimated by SEC-UV using Eq. 1 where *A*_t0_ is the IgG peak area referred to the mAb solution analyzed prior to incubation (Fig. 1A) and considered as 100% (i.e., initial amount of IgG applied to the beads, 20 μg). *A*_supernatant_ refers to the area of the IgG peak in solution measured after incubation of the beads for 30 min at 4 °C (Fig. 1B). The difference between these values gives an immediate view of the binding yield.

To verify the stability and proper binding of the antibody to the beads, three washing steps were performed with 500 μL of binding buffer (pH 8.0) and supernatants analyzed in SEC-UV. *A*_washes_ in Eq. 1 refers to the contribution of the washing steps in the final yield calculation, and detected areas were corrected for the dilution factor. As can be seen in Fig. 1C, however, the contribution of the washing solutions was almost neglectable over the total amount of IgG bound, demonstrating the stability of the binding between the antibody and the magnetic support. After three washing steps, no trace of IgG was detected.

1 $${\%}_{\text{bound}}={A}_{t0}-{A}_{\text{supernatant}}-({A}_{\text{washes*dilution factor}})$$

Four different batches of bio-functionalized beads were synthesized, which resulted in a mean of 0.195 μg IgG/μL beads, corresponding to high binding yields (*x̅* = 97.7%, *n* = 4) with an excellent repeatability (RSD 0.7%) as presented in Table 1.

**Table 1:** Data on anti-human procollagen II IgG immobilization on protein A magnetic beads carried out on 4 different bead aliquots

Batch	μL beads	μg IgG applied	μg IgG bound	IgG binding yield (%)	Loading capacity (μg IgG/μL beads)
1	100	20	19.5	97.6	0.195
2	100	20	19.7	98.5	0.197
3	100	20	19.4	96.9	0.194
4	100	20	19.6	97.9	0.196
Mean (± SD)			*x̅* = 19.5 (± 0.1)	*x̅* = 97.7 (± 0.7)	*x̅* = 0.195 (± 0.001)

The efficacy of cross-linking was confirmed by keeping the beads under conditions known to promote the release of the IgG from protein A magnetic beads (glycine–HCl 0.1 M, pH 2.5 at 4 °C for 15 min with agitation) [19]. However, analysis of acidic supernatants (Fig. 1D) revealed no peak related to the IgG, indicating that no antibodies were released from the bio-activated support and therefore confirming that the cross-linking process was effective.

### Recombinant antigen–antibody binding/release assay development

#### Reproducibility

After the bio-activation of the magnetic particles with the antibody, a commercial recombinant human procollagen type II antigen (Gln26-Arg266 and Gly957-Leu1487), containing both N- and C- terminus propeptides, was selected for the development of the immunocapture system. The first step in the assay design was to establish the optimal antigen/antibody ratio, which, considering the IgG immobilization yields (Table 1), was set at 2:1 (mol/mol) to saturate the binding capacity of the bio-activated beads considering a 2:1 stoichiometry. Therefore, aliquots of 25 μL of bio-activated beads were incubated with 50 μL of an r-Ag standard solution (0.04 μg/μL in PBS 1 ×). To promote the antigen–antibody binding, the solutions were maintained at 4 °C, as this temperature facilitates the formation of hydrogen bonds, which are essential for effective Ag-IgG interaction [24]. To verify that the Ag-IgG binding remained stable, and no significant loss of the antigen bound occurred, consecutive washing steps with 500 μL of PBS 1 × were performed. We found out that three washing steps were sufficient, since the RP-LC-UV analysis of the third washing solution returned in all the cases clean chromatograms (data not shown). In order to determine the optimal elution method for our assay and facilitate the release of r-Ag, various elution conditions were tested (strongly acid/basic pH; increased ionic strength; denaturing agents, i.e., urea, acetonitrile/methanol) [25]. No elution was observed in all the explored conditions, except for 0.2 M Gly-HCl at pH 2.5. Under these conditions, different incubation times (15 min, 30 min, 1 h, 2 h) were explored. As no increase in r-Ag release was observed, elution was set at 15 min. To test the release of the antigen under conditions that could hinder its elution, the process was performed at a temperature of 4 °C. However, comparable results were obtained by performing elution at ambient temperature as well (see Table 2, batch 4c*). Moreover, to verify that the release of the r-Ag had occurred completely, a second elution step was always repeated, and solutions monitored with RP-LC-UV.

**Table 2:** Data from the evaluation of r-Ag-mAb binding reproducibility. Elution steps of batch 4c* were carried out at 25 °C. Please refer to Table 1 for the batch numbers

Batch	*n*		Area (mAU*s)	Ag concentration (μg/μL)	Volume (μL)	Ag eluted (μg)	Total Ag eluted (μg)
2	1	Elution 1 Elution 2	317.1 33.6	0.014 0.002	50.0 50.0	0.71 0.12	0.83
4a	2	Elution 1 Elution 2	151.7 39.1	0.007 0.003	50.0 50.0	0.37 0.13	0.50
4b	3	Elution 1 Elution 2	279 4.2	0.013 0.001	50.0 50.0	0.63 0.06	0.69
4c*	4	Elution 1 Elution 2	258.1 6.0	0.012 0.001	50.0 50.0	0.59 0.06	0.65
Mean (± SD)							0.67 (± 0.14)

Quantitation of recovered bound antigen was made on the acidic eluent solutions, and a calibration curve was constructed with antigen solutions ranging from 0.006 to 0.04 μg/μL. The calibration curve obtained was *y* = 23,805*x* − 23.346 (*R*^2^ = 0.9995, mean CV% on areas 2.0%). Results are given in Table 2, where the total r-Ag released from the magnetic-based system is measured as the sum of the two consecutive elution steps. Under the reported conditions, a mean of 0.67 μg of r-Ag was proven to be captured by 25 μL of freshly bio-activated bead aliquots (*n* = 4). Considering the elution volume (50 μL), this amount provides antigen solutions of 13.4 μg/mL that were directly analyzed by RP-LC-UV without the need of any further sample preparation step. The reproducibility of the immunocapture method was also considered highly satisfactory, with RSD values of 20.89%. Bead aliquots came from two different immobilization processes (Table 1), thus allowing also for the estimation of the reliability of the preparation method.

#### Reusability and stability of the support over time

**Fig. 2:**
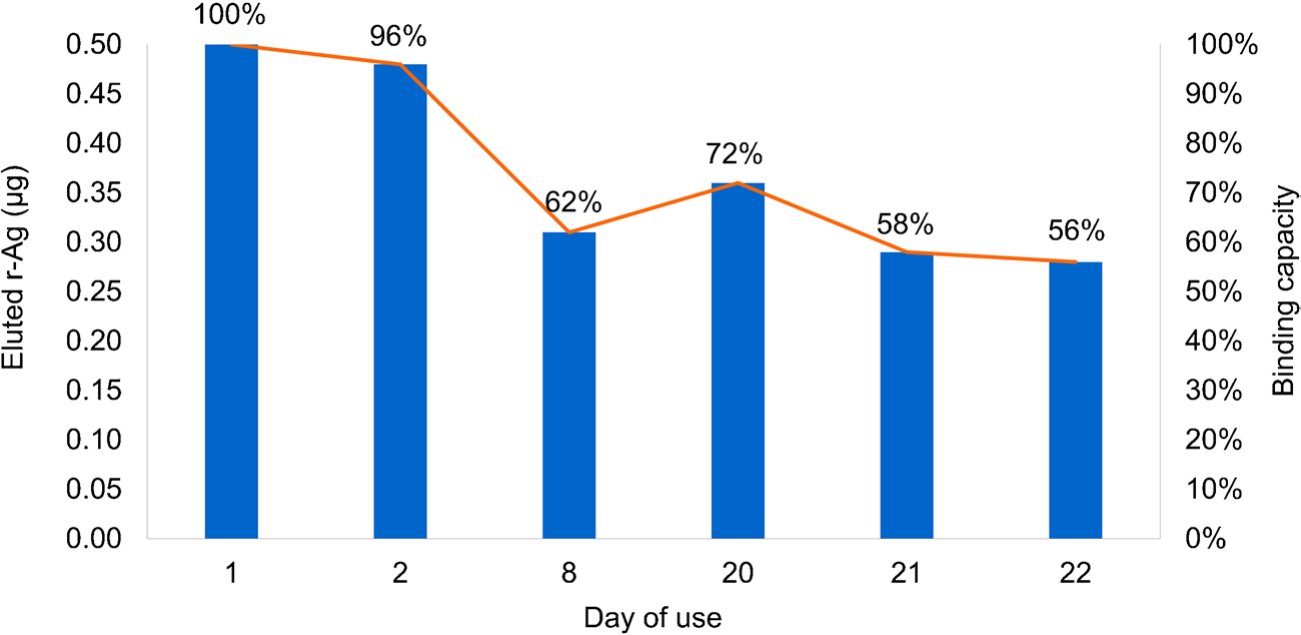
Stability of the bead-based system tested over 21 days from the first use

Reusability and stability of the bead-based system were also tested as they are important aspects to consider when designing and implementing this type of assay. The *reusability* of IgG beads was investigated by performing four r-Ag binding and release cycles on the same IgG bead aliquot (25 μL). One cycle was performed on freshly prepared beads, and the r-Ag released was considered as 100% (day 1). Then three cycles were consecutively carried out (day 2), and the overall results are reported in Table 3. The data confirmed that the same bio-activated beads can be used for repetitive immunoaffinity experiments showing the same binding and release efficiency. Furthermore, the *stability* of functionalized beads was evaluated over a period of 21 days from the initial use. The results, shown in Fig. 2, indicate that the system maintains a high level of efficiency, keeping the binding capacity at 56% after 22 days of multiple use. Recycling the bio-activated beads could contribute to a significant reduction in assay costs making this system more feasible and attractive for the processing of numerous samples.

**Table 3:** Assessment of bead reusability by 4 consecutive reuse trials on the same 25 μL bio-activated bead aliquot

Reuse trials		Ag eluted (μg)	Release efficiency (%)
Day 1	Replicate 1	0.50	100
Day 2	Replicate 2	0.51	103
	Replicate 3	0.44	89
	Replicate 4	0.50	100

#### Binding specificity

**Fig. 3:**
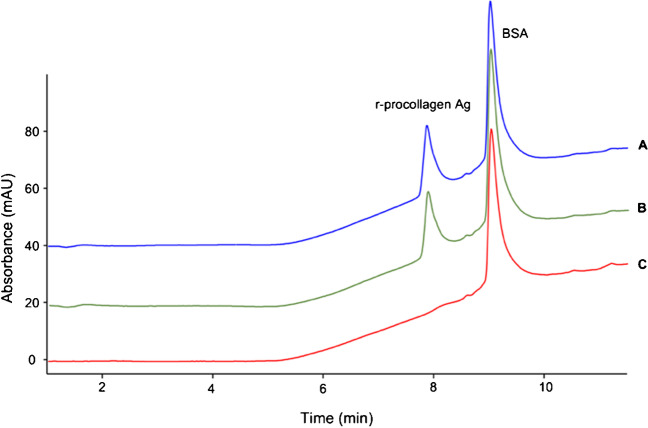
RP-LC-UV evaluation of the Ag-mAb binding specificity and the presence of non-specific interaction with the support. Mobile phases here included 0.1% formic acid. **A**, *T*_0_ solution of r-Ag and BSA (0.08 μg/μL). **B**, supernatant recovered after incubation with 25 μL of *non-functionalized beads* for 1 h at 4 °C under agitation. **C**, supernatant recovered after incubation with 25 μL of *functionalized beads* for 1 h at 4 °C under agitation

To monitor antigens in complex biological mixtures, it is crucial to ensure that the selected IgG used to prepare the bio-activated support specifically binds to the intended antigen and not to other proteins. Therefore, to test the *specificity* of the Ag-mAb binding of the system, BSA (0.04 μg/μL) chosen as standard protein was added to the 0.04 μg/μL r-Ag solution. The mixture was analyzed with RP-LC-UV and the peak areas considered as the *t*_0_ (A_r-Ag_ = 288.3 mAU; *A*_BSA_ = 899.7 mAU) (Fig. 3(A)). Then, the solution was incubated with 25 μL of bio-activated beads according to the previously developed protocol. RP-LC-UV analysis of the supernatant recovered after incubation (Fig. 3(C)) showed the disappearance of the r-Ag peak only (RT = 7.86 min), while the BSA peak (RT = 9.00 min) remained unchanged (*A*_BSA_ = 896.6 mAU), thus demonstrating the selectivity of the binding between the anti-procollagen IgG and its r-Ag. This aspect is of utmost importance not only to guarantee the specific binding, but also to avoid the saturation of the immunoaffinity material by undesired proteins, hindering the interaction with the antigen itself.

Additionally, the absence of non-specific interactions with the magnetic support was also evaluated by incubating the same r-Ag and BSA solution with 25 μL of non-functionalized beads as showed in Fig. 3(B). The presence of both peaks with unchanged areas compared to the *t*_0_ solution (*A*_r-Ag_ = 287.0 mAU; *A*_BSA_ = 901.2 mAU) led to the conclusion that proteins do not interact non-specifically with the support, as this might interfere with the system ability to accurately detect the Ag giving errors in quantitative estimations.

### Application of IgG-bead-based immunocapture system to yeast fermentation samples

**Fig. 4:**
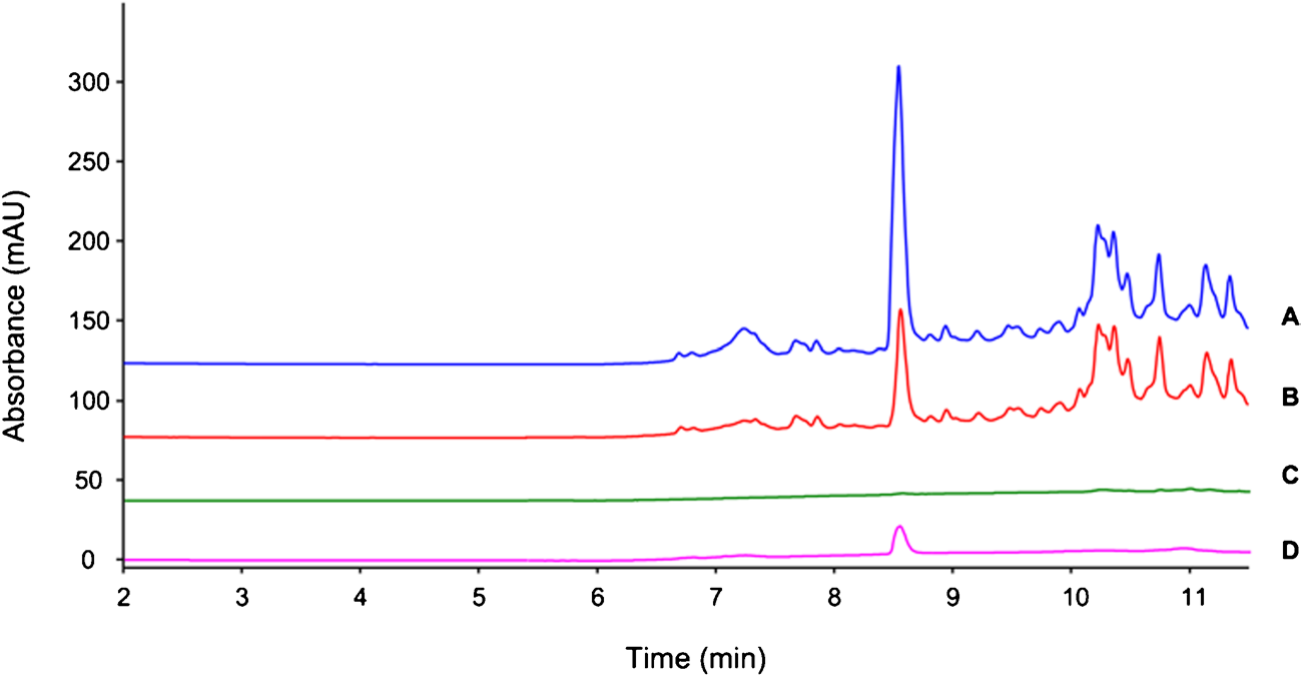
Binding and release cycle on *Pichia pastoris* fermentation sample A added with the r-Ag. **A**, 0.04 μg/μL sample A before incubation (*t*_0_). **B**, supernatant recovered after incubation. **C**, fourth washing step with 500 μL PBS 1 × . **D**, elution with 50 μL of 0.2 M glycine–HCl, pH 2.5

To provide a proof of concept of the applicability of the bio-activated bead-based system, a lyophilized sample derived from a wild-type *Pichia pastoris* fermentation process was selected and reconstituted with PBS 1 × . As this kind of biological sample is characterized by a turbid and complex nature, making it difficult to analyze as it can cause instrumentation damage, centrifugation and filtration were initially employed to observe its UV trace and to optimize all stages of the immunocapture cycle. After centrifugation and filtration, the lyophilized sample derived from the wild-type fermentation process was enriched with our r-Ag standard (sample A), and the full binding and release assay was performed as reported in Fig. 4. The antigen/antibody ratio previously tested was maintained during the experiment, with 25 μL bio-activated beads being used for the incubation of 50 μL of a 0.04 μg/μL r-Ag solution. This allowed us to compare the results to the initial conditions and to confirm the effectiveness of the system in fishing the r-Ag in real samples. A and B of Fig. 4 depict the chromatograms of the solution before and after incubation with bio-activated beads, respectively. The r-Ag peak can be seen at RT = 8.553 min in both figures, but its area is visibly reduced in Fig. 4(B), indicating the establishment of the binding with the IgG. When dealing with such complex samples, it is important to perform quantitative washings steps before promoting the release of the Ag. Four washing steps using 500 μL of PBS 1 × were set, as the fourth step resulted in a clean chromatogram as shown in Fig. 4(C). The elution was successfully promoted (Fig. 4(D)) by using previously described acidic conditions.

After setting up the immunocapture system on sample A, the binding and release assay was repeated on the raw sample (sample B) by applying the same conditions but avoiding centrifugation and filtration to reduce as much as possible sample manipulation steps. In this case, given the turbid nature of the fermentation medium and to prevent damage to the HPLC system, it was possible to analyze only the fourth washing step and the r-Ag elution step, providing chromatographic information only about the final part of the process. A bind-and-release cycle was initially carried out using the same concentration ratios. Then, three consecutive incubations were performed employing the same absolute amount of Ag applied to the beads (2 μg) but increasing the solution volume to 500 μL. The results presented in Table 4 support the ability of bio-activated beads to effectively bind the target protein (average amount eluted from the beads = 0.19 μg, SD = 0.06, RSD% = 32.3). The total amount of antigen released from the beads is lower than the value obtained for pure r-Ag in the optimization of the immunocapture assay, probably due to the biological matrix effect. This decrease in recovery value can be attributed to the turbid and suspended nature of such kind of sample, which may interfere with the optimal binding capacity of the beads. Notwithstanding this, the developed immunocapture method is reproducible and effective in isolating an adequate amount of sample for any subsequent structural characterization. Interestingly, the same capture efficiency was estimated in tenfold less concentrated raw fermentation samples.

**Table 4:** Data from *n* = 4 binding and release cycles with raw *Pichia pastoris* sample (sample B)

Batch	*n*		Area (mAU*s)	Ag concentration (μg/μL)	Volume (μL)	Ag eluted (μg)	Total Ag eluted (μg)
1 (50 μL)	1	Elution 1 Elution 2	23.6 11.1	0.002 0.001	50.0 50.0	0.10 0.07	0.17
2a (500 μL)	2	Elution 1 Elution 2	32.2 4.4	0.002 0.001	50.0 50.0	0.12 0.06	0.17
2b (500 μL)	3	Elution 1 Elution 2	83.6 5	0.004 0.001	50.0 50.0	0.22 0.06	0.28
2c (500 μL)	4	Elution 1 Elution 2	44.5 n.d.	0.003 n.d.	50.0 50.0	0.14 n.d.	0.14
Mean (± SD)							0.19 (± 0.06)

*n.d.* not detected

### RP-LC-HRMS epitope peptide mapping study

To support and validate the specificity of the proposed immunocapture method, the Ag-mAb amino acid binding region, which was not provided by the manufacturer, was experimentally determined with an RP-LC-HRMS peptide mapping study. Since the immobilized mAb is specific for human procollagen type II, it is likely that the epitope can be found on the N-terminus or C-terminus regions (propeptides), exclusive of procollagen.

An r-Ag solution was therefore digested with trypsin, and the resulting peptides were incubated with the bio-activated magnetic beads. Washes and elution steps were performed according to the protocol optimized for the intact r-Ag, and collected supernatants were analyzed using RP-LC-HRMS.

**Fig. 5:**
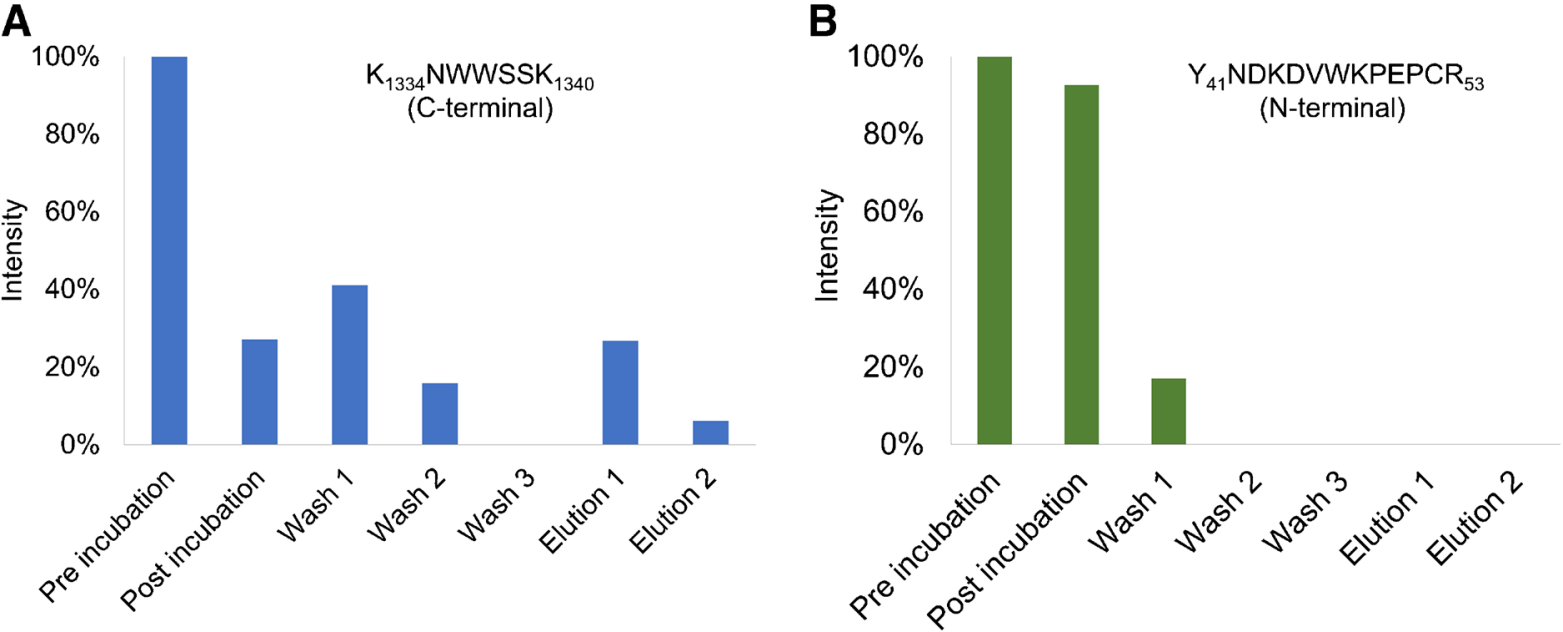
Intensity of digested r-Ag peptides through all the phases of binding and release immunocapture assay. **A**, K_1334_NWWSSK_1340_ detected C-terminal peptide (epitope). **B**, Y_41_NDKDVWKPEPCR_53_ detected N-terminal peptide (negative control)

The r-Ag digested solution was analyzed before incubation revealing 148 peptides (amino acid sequence coverage = 85.9%). After three washing cycles, carried out to remove unbound peptides, the elution step under acidic conditions was carried out to recover specifically bound peptides. In this fraction, a single peptide (seq. K_1334_NWWSSK_1340_) was detected which belongs to the C-terminus propeptide. Indeed, as shown in Fig. 5(A), its intensity decreased in the recovered supernatant after incubation with bio-activated beads compared to the pre incubation solution, indicating a successful binding interaction. The intensity of the peptide decreased further during the washing steps until it became undetectable in the final wash. In the elution steps, its intensity returned to increase, confirming that it was specifically retained by the beads. A negative control peptide (N-terminal, seq. Y_41_NDKDVWKPEPCR_53_) representative of the behavior of unbound peptides is reported in Fig. 5(B).

These experimental results allowed us to confirm the C-terminal peptide involvement in the epitope region and thus validate the specificity of the immunocapture system for human type II procollagen. These findings are also supported by literature [26].

## Conclusion

In this work, we present an original analytical method for the selective isolation of recombinant human procollagen type II, suitable for application on complex fermentation samples. This method relies on the use of magnetic beads bio-functionalized with a human anti-procollagen II antibody cross-linked via its Fc section to the magnetic support. By specifically isolating procollagen from broths, releasing it, and analyzing it, it is possible to verify if it has been produced effectively in its entirety, confirming that expression has been successful.

The immunocapture system has been found to have several advantages, including high antibody grafting, specificity in the mAb-Ag binding, and absence of non-specific protein interactions with the magnetic support. The preparation of multiple aliquots of bio-functionalized magnetic beads is a moderately time-consuming method (few hours) with affordable costs. Moreover, the reproducibility of procollagen binding was highly satisfactory, with a 21% RSD on released r-Ag from different lots of bio-activated beads. The possibility of reusing the antibody over time due to cross-linking has been proven over 22 days of repetitive use of the same mAb-magnetic bead aliquot, maintaining 56% of the binding capacity.

This easy magnetic mechanism of isolation could make the proposed analytical setup a valuable tool to semi-quantitatively monitor the state of collagen production during fermentation and could be successfully applied to other biopharmaceuticals as well.
